# Insulin resistance and high molecular weight adiponectin in obese and non-obese patients with Polycystic Ovarian Syndrome (PCOS)

**DOI:** 10.1186/s12902-021-00710-z

**Published:** 2021-03-09

**Authors:** Farnaz Kamali Haghighi Shirazi, Zohre Khodamoradi, Marjan Jeddi

**Affiliations:** 1 Department of Internal Medicine, Shiraz University of Medical Sciences, Shiraz, Iran; 2 Geriatric Research Center, Shiraz University of Medical Sciences, Shiraz, Iran; 3 Endocrinology and Metabolism Research Center, Nemazee Hospital, Shiraz University of Medical Sciences, Shiraz, 71345-1414 Iran

**Keywords:** Polycystic ovary, Insulin, Adiponectin, Obesity

## Abstract

**Background:**

Polycystic ovarian syndrome (PCOS) is the most common endocrinopathy among young women. Insulin resistance is a key feature in the pathogenesis of PCOS; also high molecular weight adiponectin is a marker of insulin resistance. The aim of this study was to evaluate the insulin resistance, metabolic and androgenic profiles and high molecular weight adiponectin in obese and non-obese PCOS patients.

**Methods:**

In this cross-sectional study in outpatient endocrinology clinics of Shiraz University of Medical Sciences, 80 women aged 17–43 years old with PCOS were enrolled. Biochemical and hormonal assay was done on fasting blood sample on the third day of follicular phase.

**Results:**

The individuals had a mean age of 28.39 ± 6.56 years, mean weight of 65.41 ± 12.59 Kg, mean BMI of 25.5 ± 4.9, and mean waist circumference of 88.0 ± 13.1 cm. Of all individuals 20% had frank insulin resistance with HOMA-IR > 3.8. Although the obese PCOS patients had lower levels of high molecular weight adiponectin (*P* = 0.03) than the normal weight PCOS individuals, the level of insulin and insulin resistance was not different in them (*P* = 0.13, 0.13). Patients with classic PCOS phenotype significantly had higher levels of insulin resistance and free androgen index (*P* **<** 0.001, 0.001). We found a significant correlation between the insulin level and free androgen index (correlation coefficient: 0.266 and *P* = 0.018) after adjusting for BMI.

**Conclusion:**

This cross-sectional study showed a high incidence of insulin resistance in PCOS patients independent of obesity, and determined BMI related lower level of high molecular weight adiponectin in obese PCOS individuals. More detailed studies are warranted for evaluation of insulin resistance and its pathophysiologic role in PCOS.

## Background

Polycystic ovarian syndrome (PCOS) is the most common endocrinopathy among women in the reproductive age. The prevalence of PCOS varies between 10 and 15% [1]. Three criteria are used to define PCOS over the last decades: The National Institutes of Health (NIH) criteria, Androgen Excess and PCOS Society (AES-PCOS) criteria, and The Rotterdam criteria [2–4].

Obesity, hyperinsulinemia, and insulin resistance are the most important metabolic abnormalities that affect PCOS patients. Hyperandrogenism, menstrual dysfunction, infertility, and hirsutism are the most common clinical symptoms in women with PCOS. In addition, the prevalence of dyslipidemia, hypertension, type 2 diabetes, and cardiovascular diseases is higher in these patients than general population [5, 6].

Although the etiology of PCOS is still not well known [1], it seems that insulin resistance is a major pathophysiologic mechanism in developing clinical symptoms and other metabolic complications of PCOS [7]. Resistance to insulin leads to an increase in free androgen availability, and consequently alteration in follicular development and granulosa cell function [5, 8]. Increased insulin concentration in PCOS patients reduces the serum level of SHBG, which enhances the bioavailability of free testosterone level; also, these women have abnormal gonadotropin concentration and great androgen biosynthesis from the adrenal and ovaries, stimulated by high level of insulin [9].

Insulin resistance has a main role in the pathogenesis of PCOS independent of obesity [10], and there is possibility of considerable effect of hyperandrogenism on the insulin resistance in PCOS patients [11].

Various adipokines are secreted from the adipose tissue, which have different effects on insulin resistance. Some of these, such as visfatin can activate the insulin receptor and has insulin-like activity, but adiponectin has insulin-sensitizing effect [12]. Adiponectin, secreted from the adipocyte, is an abundant protein that exists as multimers including high molecular weight (HMW), medium-molecular-weight, and low-molecular-weight oligomers [13]. Although some studies showed the relationship between adiponectin and PCOS independent of BMI [13, 14], some other demonstrated that the adiponectin levels were negatively associated with BMI [1, 15]. These adipokines may be considered as markers of insulin resistance in PCOS patients irrespective of BMI.

We performed this study to evaluate and compare the level of insulin, insulin resistance, androgen, and HMWA between obese and non-obese women with PCOS and in four different PCOS phenotypes; in addition, we investigated the relationship between HMWA and the androgen level.

## Methods

### Subjects

The present study is a cross sectional investigation conducted from March 2016 to February 2017 in outpatient endocrinology clinics of Shiraz University of Medical Sciences in the south of Iran. Eighty women aged 17–43 years old diagnosed with PCOS (according to the Rotterdam criteria) [4] were enrolled in the study. Pregnant women, women with late onset congenital adrenal hyperplasia, hyperprolactinemia, hypertension, diabetes mellitus, Cushing syndrome, adrenal and ovarian tumor, and those using oral contraceptive pills, lipid lowering, antihypertensive, and antiandrogen agents were excluded.

A physician measured the participants’ weight and height. A standard scale was used to the nearest 0.1 kg (Seca, Germany), while the participant was wearing light clothing and was barefoot. We measured height to the nearest 0.5 cm with a wall-mounted meter while the participant was standing with no shoes. We also calculated the BMI by dividing the weight (in kilogram) by height per square meter. Finally, we measured the waist circumference to the nearest 0.1 cm just above the patient’s uppermost lateral border of the right ilium.

The patients were divided into 3 groups based on their BMI (less than 25, between 25 and 30, more than 30 Kg/m^2^). Systolic and diastolic blood pressure (BP) was measured in one arm in a sitting and standard position and recorded.

The patients were categorized into four different phenotypes according to their androgen level, history, and sonography. Phenotype 1: chronic anovulation and polycystic ovaries but no clinical or biochemical hyperandrogenism; phenotype 2: hyperandrogenism and polycystic ovaries but ovulatory cycles; phenotype 3: hyperandrogenism and chronic anovulation but normal ovaries; and phenotype 4 (classic phenotype): hyperandrogenism, chronic anovulation, and polycystic ovaries [16].

### Biochemical and hormonal assessment

Blood samples were obtained directly from all subjects from a cannulated vein on the third day of follicular phase after 8 h overnight fasting between 8:00–9:00 AM. We performed biochemical analysis for each individual. Fasting blood glucose, triglyceride, high-density lipoprotein (HDL) cholesterol, Insulin level (Insulin -I^125^- IRMA kit, Hungary), Thyroid Stimulating Hormone (TSH), Prolactin, Luteinizing Hormone (LH) (I^125^-hLH IRMA kit, Hungary), Follicle-Stimulating Hormone (FSH) (I^125^- hFSH IRMA kit, Hungary), 17(OH) progesterone (ELISA, Germany), total testosterone (Microplate Enzyme Immunoassay kit, USA), Sex Hormone Binding Globulin (SHBG) (SHBG I125 IRMA, Hungary), free androgen index [(testosterone level/ SHBG)*100],dehydroepiandrosterone-sulfate (DHEAS) (DHEA-SO4 /I^125^ Kit, Hungary), and High-molecular-weight adiponectin (HMWA) (quantitative sandwich enzyme immunoassay technique, USA) were measured. Insulin resistance was calculated by the homeostasis model (HOMA-IR) with the following formula: HOMA-IR = [fasting glucose (mg/dl) × fasting insulin (μU/ml))/22.5] and HOMA-IR index > 3.8 was considered as “high” with clear correlation with insulin resistance [17].

### Statistical analysis

Kolmogorov-Smirnov test was performed for normality of all continuous variables. The qualitative and quantitative data were described by frequency (percentage) and mean ± standard deviation (SD), respectively. Differences between the groups were evaluated using Student’s t-test and ANOVA for the quantitative variables with normal distribution, and Mann-Whitney U and kruskal-Wallis test for the variables without normal distribution. Pearson correlation was conducted to determine the relationships between the variables. All statistical analyses were performed using SPSS software version 22 for windows. A *P*-value < 0.05 was considered statistically significant.

## Results

Data were collected from 80 PCOS individuals with a mean age of 28.39 ± 6.56 years, mean weight of 65.41 ± 12.59 Kg, mean waist circumference of 88.0 ± 13.3 cm, and mean hip circumference of 100.4 ± 11.5 cm. We found a mean BMI of 25.5 ± 4.9 kg/m^2^ (in the overweight range) in these patients. From these subjects, 39 (48.7%) had normal weight (BMI ≤24.9 Kg/m^2^), 26 (32.5%) were overweight (BMI = 25–29.9 Kg/m^2^), and 15 (18.8%) were obese (BMI ≥ 30 Kg/m^2^).

Mean level of HOMA-IR in these PCOS patients was 2.46 ± 1.30; 16 (20%) had frank insulin resistance with HOMA-IR > 3.8, and 15 (18.8%) had HOMA-IR 2.6–3.8.

From these individuals, 42.5% had PCOS phenotype1, 21.3% PCOS phenotype 2, 6.3% PCOS phenotype 3, and 30% of them had classic form of PCOS (phenotype 4). Table 1 shows anthropometric and biochemical measures in these four phenotypes.

**Table 1:** Anthropometric and biochemical measures in four PCOS phenotypes (mean ± SD and median in brackets)

Variable	PCOS phenotype 1	PCOS phenotype 2	PCOS phenotype 3	PCOS phenotype 4	***P***-value^*^
**Weight (Kg)**	64.3 ± 13.4 (62.0)	63.0 ± 9.9 (63.0)	61.0 ± 4.1 (61.0)	70.0 ± 13.6 (68.5)	0.23
**Height (cm)**	160.3 ± 6.5 (160.5)	160.3 ± 5.0 (160.0)	159.2 ± 3.3 (159.0)	159.2 ± 5.9 (158.5)	0.87
**BMI (Kg/m**^**2**^**)**	24.7 ± 4.5 (23.8)	24.7 ± 5.0 (23.0)	24.1 ± 1.6 (24.5)	27.6 ± 5.6 (26.5)	0.11
**Waist Circumference (cm)**	86.8 ± 14.0 (85.0)	82.8 ± 9.3 (83.0)	84.6 ± 10.3 (80.0)	94.1 ± 13.9 (94.5)	0.04
**Hip Circumference (cm)**	101.7 ± 10.9 (100.5)	94.6 ± 10.6 (97.0)	95.4 ± 6.6 (96.0)	103.7 ± 12.5 (103.0)	0.05
**Fasting glucose (mg/dl)**	89.7 ± 9.7 (88.5)	92.1 ± 6.1 (90.0)	92.4 ± 4.3 (94.0)	92.2 ± 6.3 (92.5)	0.13
**Total cholesterol (mg/dl)**	170.6 ± 50.6 (170.0)	167.9 ± 40.5 (173.0)	181.4 ± 18.6 (187.0)	170.0 ± 28.6 (169.0)	0.69
**HDL-cholesterol (mg/dl)**	44.7 ± 9.5 (46.0)	43.8 ± 9.0 (44.0)	47.2 ± 5.6 (47.0)	41.7 ± 9.7 (41.5)	0.53
**LDL-cholesterol (mg/dl)**	72.2 ± 33.3 (71.0)	68.5 ± 18.4 (68.0)	73.0 ± 23.2 (72.0)	69.0 ± 18.5 (70.0)	0.97
**Triglycerides (mg/dl)**	113.6 ± 65.2 (99.0)	126.5 ± 72.9 (96.0)	100.2 ± 28.2 (92.0)	133.4 ± 70.8 (127.0)	0.54
**Insulin level (μIU/mL)**	7.8 ± 3.2 (7.7)	8.7 ± 4.1 (7.5)	10.7 ± 6.5 (7.5)	16.6 ± 4.1 (16.5)	< 0.001
**HOMA-IR**	1.7 ± 0.8 (1.7)	2.0 ± 0.9 (1.8)	2.4 ± 1.5 (1.7)	3.8 ± 1.0 (3.8)	< 0.001
**TSH (ng/dl)**	3.0 ± 1.7 (2.7)	2.8 ± 1.8 (2.5)	3.2 ± 2.3 (3.2)	3.8 ± 2.5 (3.2)	0.53
**Prolactin (ng/ml)**	11.6 ± 10.1 (8.4)	6.3 ± 3.2 (5.3)	6.8 ± 4.1 (5.0)	9.5 ± 8.4 (7.1)	0.34
**LH (mIU/ml)**	4.8 ± 4.0 (4.0)	5.7 ± 3.6 (5.0)	4.9 ± 1.8 (4.9)	6.7 ± 5.6 (4.6)	0.30
**FSH (mIU/ml)**	6.4 ± 2.2 (6.6)	6.5 ± 2.6 (5.8)	9.2 ± 3.8 (9.0)	6.5 ± 2.1 (6.3)	0.10
**17(OH) progesterone (ng/ml)**	1.3 ± 0.9 (1.0)	1.3 ± 0.7 (1.1)	1.3 ± 0.7 (1.1)	2.4 ± 3.3 (1.5)	0.12
**Total Testosterone (nmol/l)**	0.5 ± 0.2 (0.4)	0.5 ± 0.2 (0.4)	0.4 ± 0.3 (0.4)	0.7 ± 0.2 (0.7)	0.009
**Free Androgen Index**	1.4 ± 0.9 (1.3)	1.2 ± 0.5 (1.1)	0.7 ± 0.6 (0.5)	2.7 ± 2.2 (2.1)	0.001
**DHEAS (μmol/l)**	2.9 ± 1.5 (3.0)	2.8 ± 1.8 (3.0)	2.7 ± 1.2 (2.5)	3.9 ± 2.0 (3.8)	0.12
**SHBG (nmol/l)**	54.5 ± 57.2 (35.0)	44.5 ± 20.0 (41.0)	71.0 ± 43.8 (63.0)	36.7 ± 23.0 (30.0)	0.07
**HMWA (ng/ml)**	2986.3 ± 1824.4 (2476.5)	2628.5 ± 1098.9 (2390.0)	2359.4 ± 1365.7 (1816.0)	2798.0 ± 1813.7 (2372.0)	0.88

*HDL* High-Density Lipoprotein, *LDL* Low-Density Lipoprotein, *HOMA-IR* Homeostatic Model Assessment – Insulin Resistance, *TSH* Thyroid Stimulating Hormone, *LH* Luteinizing Hormone, *FSH* Follicle Stimulating Hormone, *DHEAS* DeHydroEpiandrosterone Sulfate, *SHBG* Sex Hormone Binding Globulin, *HMWA* High Molecular Weight Adiponectin. Free Androgen Index: (Testosterone /SHBG)*100

* Difference between mean level, analyzed by ANOVA for normally distributed variables and kruskal-Wallis test for variables without normal distribution

Although the individuals with PCOS phenotype 4 had a higher mean BMI than other phenotypes, this difference was not statistically significant (*P* = 0.11). We found a significant difference in the waist circumference that was higher in patients with classic PCOS phenotype (*P* = 0.04).

**Fig. 1:**
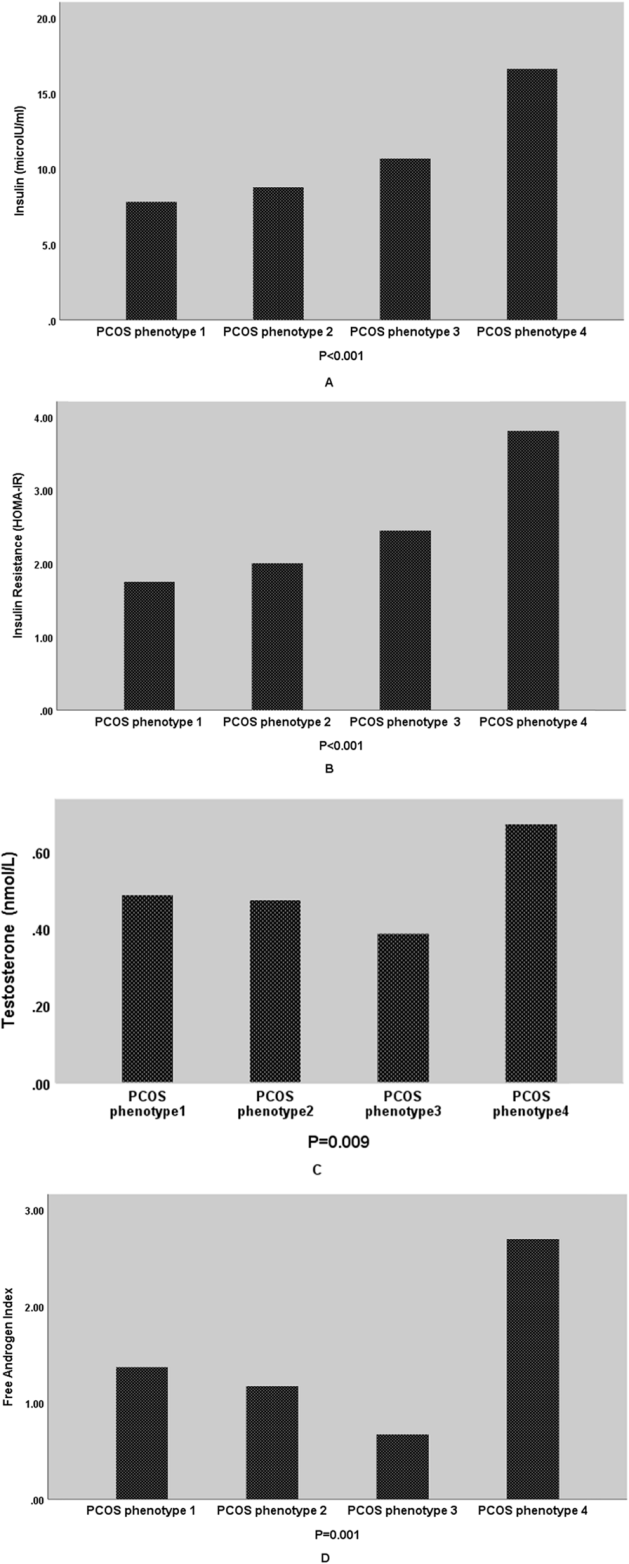
Mean level of Insulin (**a**), Insulin Resistance (**b**), Testosterone (**c**), and Free Androgen Index (**d**) in four groups of individuals based on PCOS phenotypes

By comparison of hormonal profile of the participants in the four phenotypes, we found a significant difference in the insulin level and insulin resistance that was higher in patients with classic PCOS phenotype (both *P* < 0.001). In addition, we found a greater level of testosterone and free androgen index in classic PCOS patients (*P* = 0.009 and 0.001). Figure 1 shows these data in simple bars.

We did not find a significant difference in HMWA among the four PCOS phenotypes (*P* = 0.88 by kruskal-Wallis test).

Table 2 shows biochemical parameters in all participants and separately in different BMI groups.

**Table 2:** Biochemical measures of all study subjects and separately in normal weight, overweight, and obese PCOS individuals (mean ± SD and median in brackets)

Variable	All participants	BMI groups			
		< 25	25–29.9	≥30	***P***-value^*^
**Fasting glucose (mg/dl)**	91.19 ± 7.83 (90.0)	90.59 ± 6.10 (90.0)	92.62 ± 10.34 (92.0)	90.27 ± 6.97 (89.0)	0.65
**Total cholesterol (mg/dl)**	170.55 ± 40.85 (172.0)	170.15 ± 50.59 (173.0)	177.23 ± 26.18 (174.0)	160.00 ± 32.53 (162.0)	0.37
**HDL-cholesterol (mg/dl)**	43.78 ± 9.28 (44.0)	44.72 ± 9.10 (46.0)	43.54 ± 11.12 (42.0)	41.73 ± 5.73 (43.0)	0.57
**LDL-cholesterol (mg/dl)**	70.56 ± 25.76 (70.0)	72.36 ± 32.73 (70.0)	71.27 ± 64.21 (70.0)	64.67 ± 21.02 (63.0)	0.46
**Triglycerides (mg/dl)**	121.48 ± 66.81 (101.0)	109.08 ± 61.67 (91.0)	147.04 ± 81.56 (125.0)	109.40 ± 34.45 (113.0)	0.04
**Insulin level (μIU/mL)**	10.84 ± 5.43 (9.2)	9.39 ± 4.47 (8.2)	12.17 ± 6.10 (12.7)	12.33 ± 5.88 (11.0)	0.13
**HOMA-IR**	2.4 ± 1.3 (2.0)	2.13 ± 1.10 (1.8)	2.81 ± 1.45 (3.0)	2.76 ± 1.37 (2.3)	0.13
**TSH (ng/dl)**	3.19 ± 2.02 (2.8)	3.18 ± 2.15 (2.9)	2.84 ± 1.39 (2.6)	3.85 ± 2.55 (3.0)	0.60
**Prolactin (ng/ml)**	9.54 ± 8.44 (7.6)	9.92 ± 7.64 (7.7)	10.50 ± 10.66 (8.9)	6.91 ± 5.58 (5.9)	0.38
**LH (mIU/ml)**	5.54 ± 4.37 (4.4)	6.26 ± 4.45 (4.8)	5.65 ± 5.07 (4.4)	3.51 ± 1.49 (4.0)	0.07
**FSH (mIU/ml)**	6.64 ± 2.40 (6.6)	6.81 ± 2.28 (6.8)	6.75 ± 2.35 (6.0)	6.03 ± 2.83 (6.0)	0.55
**17(OH) progesterone (ng/ml)**	1.63 ± 1.96 (1.2)	1.49 ± 0.84 (1.3)	1.97 ± 3.22 (1.1)	1.41 ± 1.02 (1.1)	0.50
**Total Testosterone (nmol/l)**	0.53 ± 0.25 (0.4)	0.56 ± 0.26 (0.5)	0.51 ± 0.26 (0.5)	0.51 ± 0.23 (0.5)	0.77
**Free Androgen Index**	1.67 ± 1.54)1.3)	1.47 ± 1.21 (1.2)	1.80 ± 2.06 (1.4)	2.02 ± 1.27 (1.6)	0.16
**DHEAS (μmol/l)**	3.18 ± 1.78 (3.2)	3.39 ± 2.09 (3.3)	3.11 ± 1.43 (3.0)	2.79 ± 1.48 (2.4)	0.45
**SHBG (nmol/l)**	48.09 ± 42.3 (35.0)	57.51 ± 53.3 (40.0)	43.86 ± 29.70 (36.0)	30.91 ± 15.54 (30.5)	0.04
**HMWA (ng/ml)**	2814.61 ± 1649.3 (2381.5)	3342.35 ± 1840.83 (2914.5)	2331.00 ± 1280.62 (1847.0)	2280.73 ± 1307.31 (1690.0)	0.03

*HDL* High-Density Lipoprotein, *LDL* Low-Density Lipoprotein, *HOMA-IR* Homeostatic Model Assessment – Insulin Resistance, *TSH* Thyroid Stimulating Hormone, *LH* Luteinizing Hormone, *FSH* Follicle Stimulating Hormone, *DHEAS* DeHydroEpiandrosterone Sulfate, *SHBG* Sex Hormone Binding Globulin, *HMWA* High Molecular Weight Adiponectin. Free Androgen Index: (Testosterone /SHBG)*100

* Difference between mean level, analyzed by ANOVA for normally distributed variables and kruskal-Wallis test for variables without normal distribution

We found that sex hormone binding globulin and HMWA were significantly lower in obese PCOS individuals (*p* = 0.04, 0.03); other hormonal parameters were not significantly different in the individuals with various BMI.

Table 3 demonstrates the correlation between anthropometric and biochemical parameters. We found a significant correlation between the insulin level and free androgen index (correlation coefficient: 0.305 and *P* = 0.006) that remained significant (R: 0.244, *P* = 0.032) after correction for weight, BMI, and waist circumference. In addition, we observed a significant correlation between the level of insulin resistance and free androgen index (correlation coefficient: 0.263 and *P* = 0.018).

**Table 3:** Pearson Correlation between different anthropometric and biochemical parameters (r and *p* value reported)

	BMI (Kg/m^**2**^)	Waist Circumference (m)	Insulin (μIU/mL)	Sex Hormone Binding Globulin (nmol/l)	Insulin Resistance (HOMA-IR)	High Molecular Weight Adiponectin (ng/ml)	Total Testosterone (nmol/l)
**Free Androgen Index**	**0.172 (0.12)**	**0.141 (0.21)**	**0.305 (0.006)**	**−0.427 (< 0.001)**	**0.263 (0.01)**	**−0.067 (0.55)**	**0.601 (< 0.001)**
**Total Testosterone (nmol/l)**	**−0.019 (0.86)**	**0.043 (0.70)**	**0.212 (0.059)**	**−0.012 (0.91)**	**0.202 (0.07)**	**0.079 (0.48)**	
**High Molecular Weight Adiponectin (ng/ml)**	**−0.275 (0.014)**	**−0.259 (0.02)**	**− 0.038 (0.73)**	**0.169 (0.13)**	**− 0.036 (0.75)**		
**Insulin Resistance (HOMA-IR)**	**0.330 (0.003)**	**0.297 (0.007)**	**0.985 (< 0.001)**	**−0.128 (0.25)**			
**Sex Hormone Binding Globulin (nmol/l)**	**−0.295 (0.008)**	**−0.205 (0.06)**	**− 0.118 (0.29)**				
**Insulin (μIU/mL)**	**0.336 (0.002)**	**0.303 (0.006)**					
**Waist Circumference (m)**	**0.763 (< 0.001)**						

We did not find any association between free androgen index with BMI or HMWA.

## Discussion

PCOS is the most common endocrine disorder of the reproductive-aged women. Over the past 30 years, it has been clearly documented that insulin resistance plays an important role in the pathogenesis of the reproductive and metabolic abnormalities of this disorder [18–21].

This study aimed to assess the importance of the insulin level and insulin resistance in PCOS patients based on obesity. We found BMI unrelated insulin resistance in PCOS individuals and a significant correlation between insulin level and free androgen index, irrespective of weight.

Human body is composed of fat mass and fat free mass. Body composition is different by sex and age and this difference is determined by the androgens level [22]. Apart from BMI, among women with PCOS, there is a high prevalence of upper-body obesity, as shown by increased waist circumference and waist-hip ratio as compared to the BMI-matched control women [23–25]. The effect of abdominal obesity on PCOS is probably greater than anticipated because this phenotype is associated with further hyperandrogenism and insulin resistance [26, 27]. In agreement with this idea, the results of this cross-sectional study showed that PCOS individuals had central obesity with a mean waist circumference of 88.0 ± 13.3; also, the patients with classic PCOS phenotype had larger waist circumference and higher level of insulin, insulin resistance, and testosterone compared to other phenotypes.

Some previous studies have investigated the impact of obesity on the hyperandrogenic state in women with PCOS. They uniformly showed higher insulin resistance, lower SHBG plasma levels, and greater evidence of hyperandrogenism in obese PCOS women compared to their normal weight counterparts [5, 8, 28]. The results of some the other studies demonstrated that insulin resistance was present in both obese and non-obese women with PCOS [29–31]. In this study, although obese PCOS individuals had lower SHBG levels, we did not find a significant difference in insulin resistance and testosterone level between obese and non-obese PCOS patients. It is well known that most PCOS patients have some degrees of insulin resistance regardless of their weight and BMI. This feature can be explained by increased phosphorylation of the serine residue of the insulin receptor substrate-1 molecule, and inhibition of insulin receptor signaling in lean PCOS individuals [32].

Moghetti et al. revealed that approximately 70% of PCOS women were insulin resistant; they also mentioned that insulin resistance and hyperandrogenism are two correlated components in the pathogenesis of PCOS. They also evaluated insulin resistance in different types of PCOS phenotypes and found that insulin resistance increased in a statically significant relationship with the severity of PCOS [33].

In this study, we observed higher waist circumference, greater level of insulin, insulin resistance, and androgen in individuals with classic phenotype of PCOS. These findings are inconsistent with the results of a previous study in a representative sample of Iranian women, which did not find a significant difference in insulin resistance and metabolic characteristics among different PCOS phenotypes [34]. This discrepancy can be the result of different study designs, and variable diagnostic criteria for classification of obesity and PCOS phenotypes in these investigations. In addition, it can suggest that there are different pathogenic pathways for different PCOS phenotypes [16].

In this cross-sectional study, we measured the level of high molecular weight adiponectin, a circulating protein produced by adipocytes. Hara and coworkers found higher predictive value of HMWA than total adiponectin for assessment of insulin resistance [35]. We observed that obese PCOS patients had lower level of HMWA. Although it seems that high concentration of testosterone in PCOS patients inhibits the secretion of HMWA by the adipocytes [36], we did not find any association between the androgen level and this adipokine. It seems that there is a complex mechanism for HMWA inhibition by androgen; also, there is debate in the relative influence of estrogens and androgens on HMWA [37].

Conor et al. in their study showed that HMWA was selectively reduced in women with PCOS, independent of BMI [13]. Our study showed that reduction in HMWA level was significantly related to BMI. This difference can be explained by different genetic characteristics and variable patterns of fat deposition in various population.

### Limitations

This study had some limitations such as small sample size and lack of BMI matched non-PCOS control group.

## Conclusion

In conclusion, our results indicate the high incidence of insulin resistance in PCOS patients independent of obesity. In addition, the findings of this study demonstrated that the patients with classic PCOS phenotype had central obesity; and higher level of insulin and insulin resistance despite lack of BMI difference with other phenotypes. These findings imply the insulin resistance as the main pathophysiologic feature in PCOS patients. More detailed studies are warranted for evaluation of insulin resistance and its biomarkers in PCOS patients independent of obesity.

## Data Availability

The datasets used and analyzed during the present study are available from the corresponding author on reasonable request.

## Abbreviations

BMI: Body Mass Index
DHEAS: De Hydro Epi Androsterone- Sulfate
FSH: Follicle-Stimulating Hormone
HDL: High-Density Lipoprotein
HMWA: High Molecular Weight Adiponectin
HOMA-IR: Homeostatic Model Assessment for Insulin Resistance
LH: Luteinizing Hormone
PCOS: Polycystic Ovarian Syndrome
SHBG: Sex Hormone Binding Globulin
TSH: Thyroid-Stimulating Hormone
